# Death and Melody: A Psychobiography of Rezső Seress, Composer of “Gloomy Sunday”

**DOI:** 10.5964/ejop.19131

**Published:** 2025-11-28

**Authors:** Virág Rab

**Affiliations:** 1 Department of Contemporary History, Faculty of Humanities and Social Sciences, University of Pécs, Pécs, Hungary; University of Johannesburg, Johannesburg, South Africa

**Keywords:** Rezső Seress, Gloomy Sunday, tragic semiperipheral self (TSS), suicide, negation, Hungary, world-systems theory

## Abstract

Rezső Seress (1899–1968), the composer of the world-famous song “Gloomy Sunday,” whose life ended in suicide, is analyzed using a psychobiographical approach. The research question is how can suicide be read through Seress’s self-narrations, embedded in the Hungarian cultural, social, and historical context? To answer this question, we integrate Kézdi’s theory of the negative code, Wallerstein’s world-systems theory, and McAdams’s narrative identity theory, linking language, structure, and the self. We analyzed nine song lyrics: counted explicit and narrowly defined implicit negations, calculated negation density (per 100 words), and coded narrative tone and plot, key life events, and agency/communion cues. Throughout the analysis, interpretation is hermeneutic and context-sensitive, with numbers serving as guides. Peaks in negation density appear in the songs “Nobody Has Ever Loved Me” (1930) and especially, “Just Drink, Drink” (1940), aligning with personal and historical crises. Agency markers are minimal or defensive, while communion is consistently framed as loss. No redemptive arc is identifiable; the life story follows a contamination sequence beginning in adversity. These patterns motivate the introduction of the tragic semiperipheral self (TSS) as a culturally and structurally situated narrative type.

*“I never lie in a song; I don’t want to write beautiful lies — I write about life, about reality in my songs, because without experience, one cannot write truthfully. I don’t look for the words or the melody — they come on their own. That’s why I can plant every single song of mine directly into people’s hearts.”*

*— Rezső Seress (Hiradó, 1936, August 27)*

## Significance and Relevance of the Topic

Rezső Seress (1899–1968), a Hungarian composer-pianist, wrote “Gloomy Sunday,” a song that achieved an international career and remains, to this day, a symbol of death and suicide in collective memory (Assmann, 1995, 2011; Halbwachs, 1992). The song functions as a cultural signifier (Bruner, 1990) linked to suicide, and both high culture (theatre, literature) and popular media use it as a reference point for loss and mourning (Patakfalvi-Czirják, 2017; Stack et al., 2008; Wertsch, 2002). Because both the song (transnationally circulating) and its composer (whose remembrance is largely domestic) have been remembered across generations — and a cult has formed around them — both have become part of collective identity. Thus, Seress’s individual tragedy — the lack of recognition, his marginalization, and his death — has entered the available collective narrative repertoire (master narrative) (Hammack, 2008; McLean & Syed, 2015; Somers, 1994). When such a template becomes a lasting part of a life story, we may speak of a self-type (McAdams, 1993, 2001, 2006). Accordingly, below we introduce the concept of the tragic semiperipheral self (TSS), a self-type tied to a sociocultural context (cf. McAdams, 2006).

Hungarian national identity has been examined by numerous scholars, with differing emphases (Csepeli, 2002; Erős, 2001; Hunyady, 1997; László et al., 2002; Pataki & Ritoók, 1999). Among these, László and colleagues assign a decisive role to collective memory and to the major historical events that shape it (László, 2008; László et al., 2002). They highlight, on the one hand, a losing narrative perspective (László, 2008), and, on the other, an emotional pattern of collective victimhood (see Bar-Tal et al., 2009; László, 2008) — both serving as background and shaping influences for the TSS (McLean & Syed, 2015). Seress lived through both world wars — which, from a Hungarian perspective, were defeats — as well as the Treaty of Trianon, a national trauma, and he also endured the Holocaust.

### Focus of the Study and Research Aims

The study is a psychobiography that focuses on understanding Seress — as an interpretive, reflective agent — (Ponterotto, 2025, p. 7), whose life ended tragically: within a short period, he made two suicide attempts. Seress is a subject from a non-WEIRD (i.e., not Western, Educated, Industrialized, Rich, and Democratic) cultural context (Mayer et al., 2023), whose career does not fall among traditionally successful life paths. Moreover, since the phenomenon and meaning of suicide are complex issues, we created an integrated theoretical framework (Fekete, 2002; Fekete & Kelemen, 1991, p. 55; Fekete & Osváth, 2004; Kézdi, 1995, p. 38; McLean & Syed, 2015): Kézdi’s theory of the negative code (Kézdi, 1995), Wallerstein’s world-systems theory (Wallerstein, 2010), and McAdams’s theory of narrative identity (McAdams, 1993). Guided by this framework, the study seeks to examine Seress’s meaning-making through his linguistic–cultural and semiperipheral structural embeddedness. In addition, through Seress’s life course and his self-destructive act, it also attempts to identify collective experiences such as suffering, loss, and marginality. Finally, to uncover Seress’s identity and interpret his suicide, it attempts to resolve the paradox arising from the tensions between his protest against the label “the song of suicides” and his own suicide.

## Theoretical and Methodological Framework

The literature describes suicide as a complex sociopsychological phenomenon (Fekete & Osváth, 2004; Zonda, 2006). Therefore — as shown in Table 1 — we also aimed to examine the psychological aspects of Seress’s self-destructive act in interaction with its cultural and social context, within an integrative (Kézdi–Wallerstein–McAdams) theoretical and methodological framework. This underscores the fact that the different aspects are in a reflexive relationship with one another — that is, they mutually shape each other (Kézdi, 1995, pp. 41–54).

**Table 1:** Theoretical Frameworks and Their Disciplinary Focus

Theory	Discipline/Approach	Focus of Analysis
Negative Code (Kézdi, 1995)	Psychology, cultural anthropology, sociology / hermeneutics	Interpretation of the cultural meaning of tragic actions
World-Systems Theory (Wallerstein)	Social sciences	Consequences of the center–periphery relationship (for identity and culture)
Context-sensitive reading of McAdams's narrative identity theory	(Narrative) psychology	Self-understanding constructed in a resilient or tragic mode

Balázs Kézdi’s “negative code” offers a culturally embedded interpretive framework through which members of a culture are able to make sense of suicide. As he puts it, “The difference between languages is not a difference of sounds and signs but of worldviews” (Kézdi, 1995, p. 55, our translation). The negative code thus structures the way suicide is conceptualized. The individual interprets this code through linguistic features — such as negation. The “negation code” is one of the linguistic markers through which the negative code becomes legible (see also Fekete, 2002) — and this phenomenon is not unique to the Hungarian language.

However, Hungarian has a feature that is absent from most other languages: the duplication of negative words (for example, instead of “nobody knows,” it says “nobody does not know”). Triple negation is also possible: “Senki(1) sem(2) mondott semmit(3)” (“No one said anything”), which, according to Kézdi, also carries cultural significance. Beyond expressing absence, multiple negation signifies finiteness. Hungarian, through negation, privileges not only impossibility but also finiteness, thereby fostering the emergence of finalistic narratives. In the case of the previous sentence, it implies that we do not even consider the possibility of any alternative to “nobody said anything”. This plays a role in the formation of one’s narrative identity as well. Thus, negation is highly represented in the Hungarian language. “Negative language use” is common even in everyday communication.

Wallerstein’s world-systems theory, which explains the functioning of the capitalist economy and highlights the unequal relationship between the center and the periphery, also offers insight into why the meaning of suicide is interpreted the way it is in the semiperiphery. His theory can serve as a tool to reveal the structural background of the Hungarian composer’s invisibility and marginalization. In particular, the cultural context of the global center–periphery relationship is suitable for understanding the “Seress phenomenon”.

Additional support for this comes from the research of Emília Barna. Barna points out that cultural values are distributed unequally at the systemic level to the disadvantage of the semiperipheral musical world. Production reaches the market through intermediary networks and platforms controlled by the center; consequently, the flows of revenue are also controlled by the center. This often entails the ‘repackaging’ of musical products originating from the semiperiphery, in order to fade the semiperipheral context (Barna, 2025). Within this framework, Seress can be seen as a victim of a musical and cultural system that is unable to cope with unprocessed pain and only allows the semiperipheral creator to benefit in a very limited way from the profit generated by the song (Barna, 2025; Barna et al., 2019).

The theories of Kézdi and Wallerstein not only complement each other but also align well with the perspective of non-WEIRD psychobiography. Both the negative code and the semiperipheral position influence how an individual’s personal narrative is constructed: Hungarian identity assumes a position in relation to the center, and this constant pressure of comparison reveals the structural background of the tragic self. In Seress’s case, it also explains why there is no redemptive narrative. The *tragic semiperipheral self* — in contrast to McAdams’s *redemptive self* (cf. McAdams, 2006) — is constructed not through redemption but through tragedy. It follows a McAdamsian *contaminative narrative*, but not by ruining something once good — it begins from an already adverse position.

Moreover, Balázs Kézdi’s theory also reveals that Seress was both a subject who experienced the negative code and, through his songs, a musical carrier and shaper of collective emotional patterns structured by that code. When applied in a psychobiographical framework, Kézdi’s theory helps us understand how this fed back into Seress’s identity and became integrated into his personal narrative.

In relation to the historical context of the psychobiography of Seress, the research of Fekete and Kelemen deserves special mention, as it focuses on the mentality history of suicide and emphasizes the historically shifting meanings of self-destructive acts (Fekete & Kelemen, 1991, p. 56). Furthermore, the study by Fekete and Osváth (2004) stresses that Hungarian culture does not offer a clearly life-affirming response to those in crisis. Because it is neither explicitly rejecting nor openly accepting, it transmits contradictory messages to members of the culture. Since self-understanding always occurs through cultural mirrors, such contradictory reflections may increase the likelihood of extreme decisions during crises. Contradictory social responses and the individual’s contradictory identity are therefore never a random coincidence, but rather mutually reinforcing relational systems (Fekete & Osváth, 2004, p. 11).

### Interpretive and Structural Frames

This psychobiography is fundamentally idiographic, examining Seress’s oeuvre, songs, and self-narratives within a specific cultural and historical embeddedness. It is also partly nomothetic — that is, generalizing — regarding the systemic absence of a redemptive narrative and the structural patterns of Central and Eastern European identity. The interaction between idiographic and nomothetic levels is enabled by investigating the interplay among three theoretical frameworks: Kézdi’s theory of the negative code, Wallerstein’s world-systems analysis, and McAdams’s theory of narrative identity. This triangulation allows us to explore identity patterns that reflect both personal and structurally conditioned characteristics. We begin with the assumption that Seress’s suicide can be understood only within cultural, linguistic, social, and historical contexts (Fekete & Kelemen, 1991; Fekete & Osváth, 2004; Kézdi, 1995). Furthermore, Hungary — at the semiperiphery, where political, economic, and social instability are persistent — contributes to the meaning of Seress’s tragic fate and explicitly self-destructive act (Wallerstein, 2010, pp. 88, 144). Therefore, it is not sufficient to search for the objective causes of Seress’s actions; we must also apprehend the narratives, linguistic codes, and metaphors that his cultural milieu employed as interpretive frameworks (Fekete & Kelemen, 1991; Fekete & Osváth, 2004; Kézdi, 1995). Rather than relying solely on the idiographic/nomothetic dichotomy, Wallerstein’s world-systems analysis offers a distinct epistemic frame for knowing social reality (Wallerstein, 2010).

## Data Collection and Analysis

Psychobiography is built on consistent systematization of data and on their critical, complex interpretation (Burnell, 2013). Within the reception history of Seress (e.g., Müller, 1983), numerous commonplaces (*topoi*) not verifiable by primary sources have entered public knowledge; therefore, source criticism has been a central methodological consideration. For source authentication, Róbert Gál’s (2010) monograph served as a key point of reference; it is based on the composer’s papers — which are no longer accessible — and reproduces several primary sources (civil registry extracts, notes, postcards, photographs) in facsimile, although it does not provide explicit references for some biographical details. Since Seress’s papers can no longer be consulted directly (the custodian has passed away and the family sold the documents), we pursued source-critical verification of contested biographical points via a targeted interview with the archivist who processed the materials (Gál, 2025). The methodological strategy followed Yin’s case study logic: the collected data (“case record”) were filtered according to themes relevant to identity formation and suicide in Seress’s oeuvre, and the selection of the song lyrics to be analyzed was carried out accordingly (Yin, 2009). The corpus contained nine song lyrics written by Seress; the inclusion criteria were: (i) unambiguous verification of authorship; (ii) identifiability of the time of composition; (iii) substantive fit to the research question.

The interpretive framework combined the triad of cultural codes (Kézdi), semiperipheral position (Wallerstein), and personal narrative (McAdams) in a theory triangulation, yielding a framework coherent with the research aim (Yin, 2009; cf. Morrow, 2005). In line with the specificity of psychobiography, we treated the song lyrics as self-narration, that is, we read them as a primary source of the subject’s inner development and meaning-making. The quantitative textometric measures (e.g., negation density) served solely as heuristic waypoints in this procedure; every numerical signal was followed by qualitative, context-sensitive close reading and the search for counterexamples (negative case analysis).

Our aim was to uncover the mechanisms and characteristics of meaning-making operating in Seress’s self-narration. Relying on Kézdi’s theory, we counted both the explicit and the implicit forms of negation: after automatic pre-processing (tokenization, lemmatization), we calculated “negation density” (the number of negation occurrences per 100 words), and then manually checked and interpreted every case in context. We treated the indicator not as causal evidence but as a compass initiating qualitative analysis.

We organized the songs around life-history-defining key episodes and, within McAdams’s framework, examined narrative tone and plot, as well as the appearance of agency and communion. We approximated agency (and its absence) by the relative proportion of first-person singular verbs expressing will or control and of phrases marking constraint; we mapped communion by the occurrence of lexemes expressing belonging and of constructions denying belonging. Each numerical signal was followed by thematic coding and the search for counterexamples, so that pattern detection would not harden into deterministic claims.

The above procedure was supplemented by thematic-level analysis: we identified the appearance of semiperipheral and macro-level codes (Wallerstein) and the recurrent motifs (lack–desire, compulsion–freedom, proximity–isolation). This multilayered reading made it possible to describe the Seress narrative coherently as one possible configuration of the “tragic semiperipheral self”.

Beyond the songs, we also included external primary sources (contemporaneous descriptions, interviews, press materials) in the analysis. These served both as data sources and as controls: we compared the patterns identified in the songs with these external observations and interpretations. The secondary sources helped reconstruct the historical contexts that decisively influenced Seress’s life course (the world wars, the economic crisis, the rise of the radical right, the Holocaust).

All findings were interpreted within the integrated theoretical framework, with particular attention to Kézdi’s theory of the negative code (linguistic–cultural codes), Wallerstein’s concept of the semiperiphery (structural position), and McAdams’s narrative psychology (life-history organizing principles). The goal was to understand — through concrete artistic self-expression (the songs) — what role culture and Hungary’s position in the world system played in shaping Seress’s inner development, and how to frame the interpretation of the self-destructive act. Accordingly, the quantitative indicators (e.g., negation density) functioned as context-sensitive waypoints supporting qualitative interpretation. Analytic decisions were made verifiable and traceable via a decision log and reflexive memos (audit trail).

## Ethical Considerations, and Author Positionality

All research decisions were guided by a commitment to preserve the subject’s human dignity and to understand his tragic fate. The study followed the ethical guidelines in force at the University of Pécs. Given that the subject died more than half a century ago, we adopted a respectful and empathic approach: we checked the reliability of secondary sources and compensated for the limited availability of primary sources by conducting an interview with the biographer who processed Seress’s legacy.

Although the researcher was born after Seress’s death, from the late 1970s onward she became familiar with — and occasionally recognized — Seress’s name and works through popular television programs and cinematic portrayals, such as *Gloomy Sunday* (Schübel, 1999) and, indirectly, *Schindler’s List* (Spielberg, 1993). The researcher’s interest in Seress was sparked by the many disturbing contradictions she has more recently encountered regarding the composer’s life path. The most salient of these is the contrast between the song’s global triumph and Seress’s marginalization.

Trained as a historian, the author reads the lyrics as a form of self-narration and treats them as narrative (subjective) truth, analytically distinct from historical fact, applying source criticism and contextualization throughout (Bálint, 2012; Elms, 1994; Schultz, 2005). From a psychobiographical perspective, data necessary to trace the subject’s inner development are of paramount importance. At the same time, this interpretive work must rest on accurate historical scaffolding; accordingly, it is thought-provoking that, although he continues to be the subject of academic studies, plays, articles, and performance evenings to this day, his year of birth is still most often incorrectly cited as 1889 — ten years earlier than the reality (1899).

Finally, it should be noted that the author writes as a member of semiperipheral Hungarian society, embedded in — and using — the Hungarian culture and language; this positionality, an emic asset but also a potential source of normalization bias, explicitly informed the interpretation of Seress’s suicidal act.

## Psychobiography of Rezső Seress (1899–1968)

Rezső Seress’s original name was Rezső Spitzer, a name inherited from his mother, as he was born out of wedlock, not from a legally recognized relationship. His mother, Blanka Spitzer, was a 20-year-old Jewish seamstress (Budapest District VIII Civil Birth Registry, 1899, Entry No. 6171), who came from a strong cultural and religious background: her father (Jakab Spitzer) did not allow her to marry Seress’s father, András Dékány, who was not of Jewish faith (Gál, 2010, p. 13). The prohibition of the marriage signified the dominance of collective norms over personal relationships and emotions — something that directly shaped Seress’s future. The absence of a father figure may have contributed to emotional instability and the experience of conditional love. This theme returned frequently in his songs, especially in connection to women, from whom love could only be hoped for in exchange for money (*One More Night*, 1924; *No One Has Ever Loved Me*, 1930). The name “Spitzer” may have served as a constant reminder of the negative emotions associated with paternal absence, which perhaps led him, in his early twenties, to adopt the stage name “Seress”. This chronologically coincided with the 1920 *Numerus Clausus* Law, which institutionalized antisemitism in Hungary by restricting access to higher education for Jewish citizens. The official name change — according to a later amendment in his birth certificate (Budapest District VIII Civil Birth Registry, 1899, Entry No. 6171) — did not occur until 1936. Moreover, being born out of wedlock and the internal experience of marginality may be interpreted as the beginning of structural exclusion.

At the cost of many sacrifices, his mother raised her two children — Rezső and his six-year-younger sister Klára — with the help of her sister, Irma. Due to her demanding work schedule, Blanka only had time for her children on weekends (Gál, 2010, pp. 17, 20). The situation of the mother and her sister reflects the structural burden placed on women in the semiperiphery: in the lower-middle class, women were responsible not only for work, housekeeping, and childcare, but, as Irma’s role indicates, also for extended family care and certain economic functions (Bódy, 2012; Gyáni & Kövér, 2001; cf. Barna, 2017, 2025).

### Family Life

Although Seress’s relationship with his mother was loving — he remained emotionally attached to her throughout his life — this maternal figure, representing emotional stability, was only partially accessible due to the cultural pressures imposed by semiperipheral norms. This, too, may have evoked ambivalent emotions in the young Seress.

Rezső maintained a lifelong and close relationship with his sister Klára (1905–1980) (Gál, 2010, p. 12). Alongside his mother, Klára served as a stable emotional anchor, which significantly shaped Seress’s narrative self.

When Rezső was nine, his mother married Béla Purgesz, a master baker from Komárom, who was a widower and had a son named Kálmán (Gál, 2010, pp. 20–21). Although their relationship was good, the stepfather did not replace the absent father figure in Seress’s life. As an adult, Seress never had children — he never became a father — meaning that the absence of the paternal role remained unresolved.

His mother’s marriage marked a completely new phase in the family’s life — one into which Seress could not fully integrate. One reason was that Klára, his sister, was allowed to remain in the family only until the age of seven, after which she was entrusted to the care of their aunt Irma, who also lived in Budapest. For Seress, this represented a renewed experience of marginality. He may have also felt that his situation was unstable, and his trust in the permanence of a safe home and his mother’s love could have been shaken. He may even have begun to doubt his own self-worth.

### Education

Klára began school in Budapest and became a student of István Tomka, a renowned music teacher. Years later, she would be the one to transcribe Seress’s compositions (Gál, 2010, p. 24). For Seress, his sister’s continuous professional support may have offered a path out of marginality — yet at the same time, it also underscored the cultural gap between them. He sensed that music, which had always been close to him, might offer a solution, a way out of marginality. At the same time, he was acutely aware of the cultural gap between his sister and himself.

Due to his lack of formal music education, Seress relied throughout his life on others to write down his songs. This created a persistent sense of unpredictability — characteristic of life on the semiperiphery — and instilled in him the inner conviction that stability is never guaranteed.

Working together with his sister may have been a positive experience of family collaboration. At the same time, he also came to realize that his constant efforts to adapt did not result in advancement, but merely in catching up — specifically, in compensating for his lack of theoretical musical knowledge. The ongoing pressure to compensate for deficiencies is itself a collective experience typical of the semiperiphery (Wallerstein, 2010).

Rezső attended a local school in Komárom, the boys' secondary school on Majláth Street (Gál, 2010, p. 25). According to the recollections of his best friend, Imre Székely Molnár, he was not a good student, but he had a good voice and ear for music, read a great deal, and was a skilled writer (Gál, 2010, pp. 26, 28; Gál, 2025). At music school, his piano teacher did not predict a great future for him (Gál, 2010, p. 29). Overall, his experiences within formal education were not positive. On the semiperiphery, talent alone was not enough.

### Creative Works

His passion for reading and creative writing pointed to an inner world he had begun to build — still under the protective wing of his family. In his free time, he frequently went to the cinema with Imre Székely Molnár — his lifelong friend — during their school years. Cinema was one of the defining modern cultural institutions of the time. His favorite actress became Pola Negri, of Polish origin, who was nearly his contemporary. Later, Seress would accompany silent films on the piano — evidence of his particularly sensitive cultural sensibility: he improvised music that matched the mood and visual rhythm of the scenes, and he was able to align it with the audience’s reactions as well.

For the student Seress, playing the piano brought pocket money — and thus a certain degree of autonomy and freedom. He experienced that, even if he could not achieve success through formal channels in the field of music, he could still do so on his own path.

After finishing school, he found work at the nearby shipyard but soon made a spontaneous decision: at the age of 17, he joined the Benneweis Circus, which was performing in Komárom at the time (Gál, 2010, pp. 33–36; Gál, 2025). He was drawn to the glamour of the circus and the free life of the performers. For him, the circus represented a kind of escape from an average life. Based on the research of Voigt (2016), it may also have offered Seress a form of legitimacy and recognition at the time.

### Life Journey Challenges

This becomes particularly significant when we consider the genre he chose: he became a trapeze artist, a role that enjoyed especially high prestige among audiences. Seress, in this sense, “aimed high” — he desired outstanding recognition, and understood that this would require risk-taking and courage.

However, three years later, at the age of 20, he suffered an accident: he fell from the trapeze and was seriously injured. The accident occurred in Vecsés, near Budapest — the longed-for center — which he could not reach with the traveling circus. Actually, he was also forced to abandon the dream of a career he had envisioned.

This fall he experienced the threshold moment of transformation: his life story took a new direction — not only did he have to choose a new path, but he also had to build a new identity. He never spoke about his time as a circus performer in later interviews. He experienced it as a failure, one that marks the origin point of the *tragic semiperipheral self*: his personal narrative had to be rewritten once again. He was once more confronted with the fact that on the semiperiphery, there is neither a secure path nor any guarantee of success.

After the end of the traveling circus period, Seress returned to Komárom in 1919 and soon moved to Budapest to live with his sister who was then living with her husband, Gyula Sztopa, at Rákóczi Street 65, on the second floor. Although moving to the capital was a significant step, it was also a cautious one, as Seress initially lived in a small room within the safety of the family environment, from where he sought his next opportunity for a breakthrough. (Fifteen years later, at the time of his marriage, he was still living at this address.) On the other hand, this room represented a small, marginal space within the large city, so Seress’s scope of movement remained limited. He was also physically constrained, as he walked with a crutch — a constant symbolic reminder of his bodily limitations.

In his early twenties, following the suggestion of Grawatsch (Bilicsi) Tivadar, who was also applying, he enrolled in Szidi Rákosi’s actors’ school (Gál, 2010, pp. 39–40). At that point, Seress was still struggling with inner uncertainty and needed external affirmation. Although enrollment in acting school was not the result of an entirely autonomous decision, it can still be considered a new identity experiment — reinforced by the fact that he registered there under the name *Seress* (Gál, 2025). He wanted to prove himself. Precisely because of this, it was a major disappointment that, after obtaining his acting diploma, he was assigned humiliatingly small roles at the Városliget Theatre. He experienced this, like his earlier acrobat career, as a failure (Gál, 2010, p. 47). He remained in marginality and invisibility. The *tragic semiperipheral self* was reinforced. He terminated his contract.

His experiences — those of cultural exclusion and restricted mobility — were intensified by the antisemitic legislation introduced in the early Horthy era, in the first years of the 1920s (Karsai, 2005).

### Accomplishments and Highlights

His first success came relatively late, at the age of 25, when his song *Just One More Night* (1924) was published by the Nádor Kálmán Music Publishing House. This marked the beginning of his musical career, even though he had never received formal training in music. While in Komárom he had been a member of the local male choir, and had played in several venues in Pest, his first serious pianist job (at the later *Kulacs* restaurant) was obtained for him by Nádor József, the son of the music publisher’s owner, who continued to support him later on (Gál, 2010, p. 48).

This was the first time he received both institutional and professional recognition — for a work that stemmed solely from his autodidactic learning and persistence. In addition, through Nádor (as a mentor), he also gained social capital, which remained essential for his success. The venue was located just behind the immensely popular New York Café and was colloquially known as “Little New York” (from 1932, officially named *Kulacs*). In its grand hall, Seress was employed as a pianist and composer in his late twenties (Gál, 2010, p. 118). He found music — his own mode of self-expression — through which he could also hope for a livelihood. This experience was identity-forming, but did not result in an absolutely stable status, so the *tragic semiperipheral self* likely continued to strengthen.

*One More Night*, as a form of self-narrative, reflects an identity without support, whose earlier dreams had collapsed. As a *tragic semiperipheral self* motif, it symbolically evokes a death wish as well (“Then I shall leave forever”). He uses words such as *pain* and *beggar*, which are classic markers of the *negative code*.

A true breakthrough in songwriting came for Seress in the early 1930s, that is, in his early thirties (Gál, 2010, p. 66). In 1930, at the age of 31, he composed both the lyrics and music for the song *No One Has Ever Loved Me* (*Engem még nem szeretett senki*), rich in linguistic negations. In 1932, he wrote the lyrics to *Come, My Dog Bodri* (*Gyere Bodri kutyám*). During the Great Depression, the revenues at Kulacs plummeted, and the owner, Béla Vágó, tried to compensate by “commissioning” Seress to write a more positive song. The result was the seemingly positive *Let’s Love Each Other, Children* (*Szeressük egymást, gyerekek*, 1932).

Unusually, *Gloomy Sunday* (*Szomorú vasárnap*) was not released by Nádor, but by the publishers Csárdás and Marnitz in 1933, who held both domestic and international distribution rights. The lyrics were written by László Jávor, a police radio reporter for the *8 Órai Újság*, who asked Seress to set them to music. The orchestration was done by Kornél Arányi, a trained musician who had studied at the Academy of Music (Gál, 2010, p. 69). This marked a step toward professionalization, as up until that point, it had been Seress’s sister who helped transcribe his music. This time, Seress was also able to translate the success into financial gain. With the advance payment for *Gloomy Sunday*, he had a fashionable suit and a new tuxedo made. This was, in his own words, “the first luxury” of his life (Gál, 2010, p. 73) — a visible marker of success that also shaped his identity. “This is my spring,” as he called March and April of 1933 (Gál, 2010, p. 75; Gál, 2025).

The contemporary media played a major role in the unprecedentedly rapid spread and international resonance of the song (Patakfalvi-Czirják, 2017). Seress himself gained widespread media attention only after the first suicides were publicly linked to *Gloomy Sunday*. A feature article in *A Mai Nap (1935, p. 7)* quoted a line from their interview with him as a subtitle: *“Whoever dies with a song must have a beautiful soul, but I never wanted this kind of publicity”.* The interview revisited his earlier career. He spoke positively about his transition from acting to songwriting and listed his successful songs, concluding that “these songs are known by everyone — even abroad”. This statement reflects a kind of strengthened cultural self-confidence — an important element of his narrative self-understanding. He had come to recognize that what he created had value in the eyes of others, and that this recognition extended beyond the listeners who personally connected with his music.

The subject of financial recognition also came up in relation to international success: “A fashionable songwriter like me could easily earn three to four thousand pengő a month abroad just from royalties, without needing to perform publicly — I’m just happy I can live in peace like this”. This reflects a typically semiperipheral narrative: comparing one’s own situation with that of artists living in the Western center. According to Barna (2025), this is a typical pattern in the music industry among semiperipheral creators — the lack of stable livelihood and visibility often translates into existential experiences different from those in the global center. This moment also reveals a tone of resignation — an acceptance of limitations.

Seress reflects on the center–semiperiphery relationship, economic and technological inequalities, and the vulnerability of the semiperiphery from the perspective of Hungarian composers:

*“Hungarian composers write songs just as good as the Americans. The secret of their success is that in America, they are fantastic at arranging music. … If a Hungarian song is recorded on gramophone in Berlin, it completely changes due to the new arrangement.”* (*Author’s translation; as quoted* *in* Müller, 1983, p. 9)

Then, addressing the consequences, he states:

*“No one buys Hungarian compositions because the audience demands English. That’s how it becomes possible for Hungarian composers to be left penniless… and for the Hungarian pengő to be sent to those ‘poor’ American composers, who already earn 50–100 thousand dollars with a single successful song.”* (*Author’s translation; as quoted in* Müller, 1983, p. 9; see also Barna, 2025)

When asked in *A Mai Nap* whether he enjoyed playing music, Seress drew an equal sign between his life and music. Music was everything to him; he emphasized that he didn’t *sing* songs but *spoke* or *performed* them, and that he often cried while doing so. This indicated a total identification with the song — performance was part of his identity. This made him emotionally authentic and compelling to his audience.

Regarding the suicides, he rejected the accusations. Using the phrase “deadly publicity,” he suggested that what was being circulated was a myth (see Patakfalvi-Czirják, 2017; Stack et al., 2008). Although *Gloomy Sunday* made him famous — through the myth of the “killer song” that lured listeners to their deaths — this myth also trapped him in a particular interpretation. He became, irrevocably, “the composer of the suicide song,” a role he could never escape.

His rising fame and popularity clashed more and more starkly with the political rightward shift of the late 1930s, marked by antisemitic laws and open support for Hitler. The conditions for pursuing the kind of career Seress had imagined for himself were increasingly undermined.

In 1934, at the age of 35, Seress married Hani (Helén) Nádler, the waitress at the Kulacs Restaurant (*Budapest District VII Civil Marriage Register, 1934*). It was around the time they met that he composed the song *When Everything Ends* (*Ha minden véget ér*). This song explicitly deals with finality and the inevitability of loss. Themes of death and transience also appear implicitly through metaphors such as “silent path,” “quiet passing,” and even hints at the closure of the narrative self: “we can no longer think about the past”. In this song, Seress addresses the woman he hopes to win over in the language of the negative code with an unusually dense pattern of implicit negations — a mode of communication that had previously brought him emotional validation and success and did so once again. By this point, the *tragic semiperipheral self* had become fully internalized. Paradoxically, his goal — winning another person’s love — was achieved through a song that emphasized endings. The newlyweds moved into a dark, courtyard-facing apartment above the Kulacs, physically merging the spaces of work and private life. From that point on, these domains were no longer separate.

In the summer of 1935, Seress returned home to Komárom, and the local press covered his visit; he was also interviewed in Pozsony (*Híradó, 1936**, p. 4*). This marked the peak of his career, yet the recognition he received remained characteristic of the semiperiphery: it was limited to local and regional attention rather than emanating from the cultural center — Budapest or the West. The spotlight remained fixed on his homeland.

### Devolution and Dissolution

Around the same time, he was also involved in a plagiarism lawsuit (filed by another composer who had written a similarly titled and themed song), concerning his work *If I Were God* (*Ha én lennék az Isten*). The case deeply affected Seress, as it cast doubt on his artistic authenticity. Since he was so intimately identified with his works, the challenge seemed to threaten his very identity. Although the Curia ultimately ruled in his favor in 1936, declaring him innocent (*Gál, 2010**, pp. 85, 87*), the ordeal took an emotional toll. Meanwhile, his operetta *Love All Along the Line* (*Szerelem az egész vonalon*) failed. It simply could not enter the elite musical space dominated by major operetta composers such as Ferenc Lehár, Imre Kálmán, and Szabolcs Fényes. Once again, Seress found himself reliving the experience of marginality.

He may have found some consolation in the fact that several of his songs became real hits during the mid-1930s, including *The Wheel Turns Up and Down* (*Egyszer fent és egyszer lent a kerék*) and *She Became Everyone’s Woman* (*Mindenki asszonya lett*). The former expressed the instability and unpredictability of life on the semiperiphery. To this body of work we can also add *The World Has Ended!* (*Vége a világnak!*), a text that responded to the historical context — war and suffering — and was set to the same melody as *Gloomy Sunday*. This song will be discussed in greater detail later.

A profound rupture — and the culmination of the *tragic semiperipheral self* — came when Helén, his wife of Jewish faith (*Budapest District VII Civil Marriage Register, 1934*), left him. Sometime after the Anschluss (March 1938) and amid the increasingly frequent enactment of antisemitic laws, she disappeared — we do not know where — and urged her husband to flee as well. Seress, however, chose to stay. It was during this time (1940) that he composed the music for *Just Drink, Drink* (*Csak inni, inni*), a song about coping with pain through self-destruction. By this point, nearly everyone had vanished from his life — even his closest friend, Imre Székely Molnár, who had emigrated to Canada. In response to this new loss, he composed the ironically entitled *Hello, You Old Rake* (*Szervusz, te vén kujon*, 1938), in which he again turned to alcohol to numb the emotional pain of losing a friend. From then on, Seress gradually — and eventually completely — withdrew into isolation.

Despite the increasingly strong antisemitism, Seress remained in Hungary even when the country joined World War II on Hitler's side. In the spring of 1942, he was conscripted into Labor Service. According to Act XIV of 1942, this was how Jews were required to fulfil their military obligations. Jewish labor servicemen were treated like prisoners of war. They were not regarded as human beings, and they were forced to carry out life-threatening tasks: they were made to bury live mines and were kept in inhumane conditions. Many perished due to the cruelty, and even those who survived suffered lasting physical and psychological injuries (Braham, 1997, pp. 317–325). There is little reliable information available regarding this period in Seress’s life. What is certain is that Seress experienced dehumanization and the immediate proximity of death. He internalized the most severe experiences of the semiperiphery and of historical cataclysm. He lost weight, his health declined, and his deteriorated condition was accompanied by hopelessness. In April 1945, upon the arrival of the first Russian tanks, he composed the song *Fizetek főúr* (“*Waiter, the bill, please”*), in which he spoke about having ruined his life. He returned home at the end of April, when the war was over. His sister’s family survived as well, but their parents, who were deported, died on the train en route to Auschwitz. In the reviving capital, one of the rooms of the Kulacs restaurant reopened by the end of April. Everything in the apartment above the Kulacs had remained in place, and Seress moved back there. For a long time, he only felt safe in the apartment and did not leave it (Gál, 2010, p. 112). At that time, he was dejected, melancholic, slow, and lacked willpower — he was depressed. His sister pulled him out of this state. His mood improved, and what also helped him rebuild his life was that Frigyes Marnitz, the publisher of *Gloomy Sunday*, informed him that a *Gloomy Sunday* Club operated overseas and that a Budapest-based ceramic artist was making a bust of Rezső Seress for them (Gál, 2010, p. 113). In other words, the myth was reborn, and Seress became a cult figure in the distant center. What was most important for him, however, was that Helén, his wife, unexpectedly returned to him. It was also at this time that the famous quote from Otto Klemperer, the chief music director of the Budapest Opera House, was entered into the well-known Seress guestbook: “Er ist kein Musiker — er ist nur ein Genie” (“He is not a musician — he is only a genius”), which, alongside being a compliment, also reinforced the feeling that had always tormented Seress: that without institutional background or formal studies, he stood outside the circle of professional musicians. Furthermore, following World War II, his songs evoking the atmosphere of the Horthy era (1920–1944) were not tolerated by the new Sovietized regime. His identity was under threat from all sides: on January 19, 1949, Szabad Nép published an article by Miklós Molnár launching an ideological campaign against artists evoking the past, and Seress Rezső’s songs were blacklisted. His network of connections also suffered a serious blow when Béla Vágó, the former owner of the Kulacs Restaurant and Seress’s benefactor and supporter, died by suicide on September 18, 1951 (Gál, 2010, p. 127).

At the height of the communist dictatorship, Seress played the piano at the Kispipa restaurant, and it was from there that he eventually retired. He did nothing but make music. “He barely slept and barely ate. He drank coffee by the liter.” (Gál, 2010, p. 130; Gál, 2025) He was afraid, and his dejection became chronic. The experiences he had during the labor service and the persecution of Jews caused symptoms consistent with trauma-related distress (Gál, 2025). At this time, his wife had become emotionally distant from him (Gál, 2010, p. 132). By living solely for his work, he became even more marginalized and even more vulnerable. He was not deeply embedded in any social network — his only living relatives were his sister Klára’s son and his wife, though they were genuinely close to him.

In December 1967, the elderly (68-year-old) Seress was forced into retirement (after an ill-considered and unfortunate decision left him without a contract) (Gál, 2010, p. 138). Having played music continuously for approximately 40 years, being stripped of his vocation plunged Seress into deep depression.

At that time, he and Helén were already living near the Kispipa restaurant (Dob Street 46b). It was during this period that the song *In My Life I Only Loved Two Women* (*Életemben csak két nőt szerettem*) was composed. As the song itself suggests — referring to his mother and his estranged wife — he and his wife had, to his regret, already grown very distant from each other. Near the end of his life, he shared the following reflections with Gyuláné Sztopa (Klára’s daughter-in-law), who was close to him:

“After my death, they’ll write beautiful things about me in the obituaries. That’s how it goes here. That more people know my songs than the man who wrote them. That I was a small, unnoticed little man, a *schlemiel*, in a small Budapest restaurant… I was little Seress, Rudi, to my close friends Dudi.” (Gál, 2010, p. 152)

Let us juxtapose this confession with the interview excerpt chosen as the study’s motto. His interview was marked by the tone of the negative code and the tragic semiperipheral self, conveying two important messages. First, complete identification with his works. Second, the notion that composing music was a kind of fated internal compulsion — something that aligns with what Jung also described:

“Whenever the creative force predominates, human life is ruled and molded by the unconscious as against the active will, and the conscious ego is swept along on a subterranean current, being nothing more than an observer of events. The work in progress becomes the poet’s fate and determines his psychic development. It is not Goethe who creates Faust, but Faust which creates Goethe.” (Jung, 1966)

That was the case in 1935, but in the final years before his death, Seress had written nothing further. As he told to Klára’s daughter-in-law, his songs (which were known) and he himself (who was not) were not truly one and the same. The tragic semiperipheral self had reached its conclusion — Seress knew he would never again play a leading role in life, and thus his marginalization on the semiperiphery had become final. As long as he worked, he was a “little man” in a little restaurant. And once he lost his job, he wasn't even that anymore.

His life, perhaps unsurprisingly, ended in suicide. On January 12, 1968, Seress Rezső, who had been hospitalized with severe injuries, hanged himself (Budapest District VIII Civil Death Register, 1968). A few days earlier, he had jumped from the window of his third-floor apartment, but that suicide attempt had not ended in death. The sources do not clearly indicate whether this first attempt occurred on Sunday the 7^th^ (perhaps as a reflection on the *Gloomy Sunday* myth) or Thursday the 11^th^.

## Historical Context and Cultural Patterns

According to the research of Fekete and Kelemen (1991), the changing meanings of suicide derive partly from the different mentalities of various eras. In Hungary, following the lost First World War and the dissolution of the Austro-Hungarian Monarchy, society underwent a transformation, as a result of which certain social groups — primarily the lower classes but also parts of the remaining gentry — could not find their place in the new social structure (Bódy, 2012). Among them was the autodidact musician, who was also excluded because of his Jewish origin, and who, due to political rightward shifts and the antisemitic environment, had no access to resources that might have supported his social mobility (Bódy, 2012). Throughout his life, he remained outside the cultural centers such as the prestigious New York Café and Restaurant (Czina, 2022); the most esteemed venue in which he performed, the “Little New York” (*Kis New York*), could at best be described as semiperipheral.

The existential questions arising from social problems and the collective sense of suffering were articulated through the dominant artistic and cultural language of the era — namely, Art Nouveau (Szabadi, 1981). Seress was socialized into this context, a cultural space in which the aestheticization of the “tragic self” became dominant. Alongside this, the suicide rate was also high (Bálint, 2016; Stack et al., 2008). Suicide became interpretable for members of the culture through numerous literary and musical works (Stack & Lester, 2009). Melancholy and a sensitivity to death represented a kind of shared language for members of contemporary society. According to Fekete and Kelemen (1991), between the two world wars in Hungary, a symbolic suicide culture developed, particularly in the gentry world and among “inadequate little people,” which the authors describe as a form of stylish withdrawal — an aesthetic and existential self-elimination. Beyond marginalization, suicidal behavior was also intensified at the societal level by historical traumas, including the collective trauma of the Holocaust, which Seress also experienced (Gál, 2010, pp. 99–119; Gál, 2025).

## Cross-Sectional Results

We analyzed nine song lyrics written by Seress for which, (i) authorship is unambiguously verified, (ii) the time of composition is identifiable, and (iii) they are connected to Seress’s life events. Since the widely known 1933 lyrics of *Gloomy Sunday* were written by László Jávor, we instead analyzed the narrative of Seress’s 1936 variant. However, we also included Jávor’s 1933 lyrics in the negation-density analysis (Table 2) as a comparator. *Let’s Love Each Other, Children* is documented to have been written on commission (to increase foot traffic of the Kulacs during the Great Depression); therefore, we treated it as a counterpoint when interpreting narrative tone, plot, agency/communion, and linkage to life events.

**Table 2:** Negation Metrics Across Seress Song Lyrics

Year	Title of the Song	Total Words	Explicit Negations Number	Negation Density^a^	Number of Explicit and Implicit Negations^b^
1924	*One More Night (Még egy éjszaka)*	122	4	3.25%	13 (10.65%)
1930	*Nobody Has Ever Loved Me (Engem még nem...)*	150	17	11.33%	30 (20%)
1932	*Let’s Love Each Other, Children (Szeressük...)*	162	5	3.09%	22 (13.58%)
1933 (Jávor)	*Gloomy Sunday (Szomorú vasárnap)*	69	1	1.45%	10 (14.49%)
1936	*The World Has Ended! (Vége a világnak!)*	81	2	5.13%	14 (17.28%)
1938	*I Love to Be Drunk (Én úgy szeretek...)*	187	19	10.16%	29 (15.51%)
1940	*Just Drink, Drink (Csak inni, inni)*	183	27	14.75%	60 (32.78%)
1945	*Check Please, Waiter (Fizetek, főúr)*	148	5	3.38%	14 (9.46%)
1957	*I’m Just a Passenger on Earth (Csak átutazó...)*	115	9	7.83%	17 (14.78%)
1962	*I’ve Only Loved Two Women (Életemben csak...)*	169	8	4.73%	20 (11.83%)

*Note*. All song lyrics were downloaded from Zeneszoveg.hu (2025).

^a^ (negation/100 words)

^b^ (negation density)

The analysis was conducted within the integrated Kézdi–Wallerstein–McAdams framework, in triangulation; we read the song lyrics as self-narration. In examining negation, we applied automatic pre-processing (tokenization, lemmatization), then checked the results by manual verification and context-sensitive interpretation. We counted both explicit and narrowly construed implicit negations — as shown in Table 2; the indicator is negation density (negations per 100 words). We treated quantitative signals as heuristic compasses; every numerical result was followed by close reading and the search for counterexamples (negative case analysis).

According to Table 2, negation density shows two peaks: in the 1930 *Nobody Has Ever Loved Me* and the 1940 *Just Drink, Drink*. At the time of the former, Seress was 31 years old and had been living for roughly ten years in the household of his sister and her husband, who enjoyed a stable, happy marriage. In our reading, this contrast — together with the everyday burdens within the macroeconomic context of the Great Depression (Pogány, 2023) — contributed to the negative-code formulation of the self-narrative. The line “Nobody has ever loved me” is repeated four times; the double (at points triple) negation together with the pronoun “me” (engem) strongly supports the life-narrative reading. The co-presence of past–present–future perspectives (“I am not allowed to be happy”) — following Kézdi (1995) — raises not only past lovelessness but also doubt about the very possibility of being loved. From the many negations one can read off the deepest desire: to be loved.

*Just Drink, Drink* (1940) is the song with the highest negation density. By this time — also in connection with the Anschluss and the first anti-Jewish laws — he suffers a personal loss as well (his wife, likewise of Jewish origin, leaves him). In the lyrics, defensive strategies appear one after another: “Just drink, drink,” “Trust no one,” and “And lie that I am happy” — that is, the suppression of emotion and the prior rejection of connection to others, and the deliberate control of self-presentation (impression management) also appears. We did not identify a redemptive arc; the focus is on forgetting. Through negation, in both songs Seress emphasizes the feeling of lack and rejection. A similar pattern is shown in the lyrics of *One More Night* and *I Am Just a Passenger*. Negation appears as a defense in *Just Drink, Drink*, *I Love to Be Drunk*, and *Check Please, Waiter*. These latter three lyrics are connected to the most difficult period of Seress’s life, to World War II and to Labor Service.

Examples for the development of the McAdamsian agency–communion pattern are shown in Table 3. Agency is characteristically present in a defensive form: first-person singular will/control is weak, while the markers of constrained agency (“must,” “cannot,” “I cannot”) are strong. Communion is mostly (self-)constrained and is framed as loss (e.g., *Nobody Has Ever Loved Me*). *Let’s Love Each Other, Children* functions as a counterpoint, explicitly signaling connection, that is, as a negative case it makes the dominant pattern more differentiated.

**Table 3:** Narrative Coding by Song

*Még egy éjszaka* (1924/Age 25) — *One More Night*	
Life-event link	Transitional period in Budapest: no stable livelihood and no relationship; seeking a breakthrough amid pronounced precarity.
Key lines	“I will leave forever”; “I will no longer be in your way”; “I won’t see you again anyway”.
Explicit negation	“*there is no other request”;* “*I will no longer be in your way”;*“*I won’t see you again anyway”*
Implicit negation / finality	“I will leave forever”; “for the last time”; “my last complaint”.
Agency – active (1st person)	“I will leave”; “I wait for you / I’ll be waiting for you”.
Agency – constraint	“I will no longer be [there / in your way]”.
Communion – presence	direct address (“come to me”), offer to pay as an attempt at connection.
Communion – absence	“I won’t see you again anyway”.
Tone / plot	tragic/finalistic; loss/constriction.
Semiperipheral codes	vulnerability; transactionalized intimacy (money).
*Engem még nem szeretett senki* (1930/Age 31) — *Nobody Has Ever Loved Me*	
Life-event link	Living for ~10 years in his sister and brother-in-law’s household; no stable relationship; daily hardships of the Great Depression.
Key lines	“Nobody has ever loved me” (×4); “I’m not allowed to be happy”; “there is no one to wait for me”.
Explicit negation	not; there is no/none; ‘not allowed / may not’.
Implicit negation	“orphan/alone”; “there is no one to wait for me”.
Agency – active (1st person)	little; mostly longing rather than action.
Agency – constraint	“must buy” (commodified kiss); “not allowed.”
Communion – presence	desire for connection.
Communion – absence	“nobody”; “orphan/alone”; “no one to wait for me.”
Tone / plot	tragic/finalistic; loss.
Semiperipheral codes	monetization of intimacy (“must be bought for base money”).
*Szeressük egymást, gyerekek* (1932/Age 33) — *Let’s Love Each Other, Children* (negative case)	
Life-event link	Commissioned by the Kulacs owner during the Depression to counter falling patronage; a deliberate counter-tone.
Key lines	“Let’s love each other, children”; “the grave closes over us”; “there is no awakening”.
Explicit negation	‘there is no’; ‘anyway/inevitably’ (fatalistic marker).
Implicit negation / finality	“terminal station,” “comes to an end,” “the grave encloses”.
Agency – active (1st person plural)	“Let us love,” “Let us make … happy”.
Agency – constraint	“life flies away anyway”.
Communion – presence	explicit affiliation (we, togetherness, love).
Communion – absence	the closing force of mortality.
Tone / plot	affiliative counter-tone; reconnection (under mortality).
Semiperipheral codes	scarcity of time; ephemeral stability.
*Vége a világnak* (1936/Age 37) — *The World Has Ended!*	
Life-event link	Era of war anxiety and political radicalization; apocalyptic public mood.
Key lines	“My heart no longer waits for or hopes for a new spring”; “Love has died!”; “The world has ended!”.
Explicit negation	not; ‘in vain’.
Implicit negation / finality	“has died,” “it’s over,” war imagery.
Agency – active	minimal (e.g., “I say my prayer”).
Agency – constraint	“I cry in vain,” “I suffer in vain”.
Communion – presence	prayer, address to the transcendent.
Communion – absence	“heartless… people,” devastation.
Tone / plot	apocalyptic, finalistic; loss/constriction.
Semiperipheral codes	helplessness amid historical cataclysm.
*Én úgy szeretek részeg lenni* (1938/ Age 39) — *I Love to Be Drunk*	
Life-event link	In the shadow of the Anschluss and anti-Jewish legislation; mounting existential anxiety, alcohol as coping.
Key lines	“then nothing hurts”; “I cannot live sober”; “I search for a better world”.
Explicit negation	does not hurt; I cannot.
Implicit negation	“words are deceitful,” “the world is treacherous”.
Agency – active (defensive)	“I avoid” (sobriety), “I search for” (a better world).
Agency – constraint	“I cannot live sober”.
Communion – presence	collective intoxication (1PL: “it lifts us to heaven”).
Communion – absence	connection substituted by alcohol.
Tone / plot	avoidant/escapist; contaminative.
Semiperipheral codes	coping via alcohol; instability.
*Csak inni, inni* (1940/ Age 41) — *Just Drink, Drink*	
Life-event link	Wife’s disappearance/departure after the Anschluss; first anti-Jewish laws; deepening isolation.
Key lines	“Trust no one”; “to lie that I am happy”; “I have no true good friend”.
Explicit negation	nobody/none; there is no; not.
Implicit negation	“(s/he) left me,” “to forget”.
Agency – active (defensive)	“to go/keep going,” “to lie”.
Agency – constraint	“let no one find out”; “I don’t care about the world”.
Communion – presence	—
Communion – absence	“I have no true good friend”; “Trust no one”.
Tone / plot	tragic/finalistic; loss/constriction.
Semiperipheral codes	isolation, mistrust, vulnerability.
*Fizetek, főúr* (1945/ Age 46) — *Check Please, Waiter*	
Life-event link	After labor service and wartime trauma; return to Budapest; period of withdrawal followed by gradual rebuilding.
Key lines	“I don’t have much time left”; “I go on without farewell”; “heavenly café”.
Explicit negation	there is no; “never good” (negated evaluation).
Implicit negation / finality	farewell, “there is no more light”.
Agency – active (limited)	“I pay,” “I hurry”.
Agency – constraint	“I ruined it (my life)”.
Communion – presence	waiting for someone; “heavenly café” as imagined sociability.
Communion – absence	farewell, departure.
Tone / plot	elegiac, finalistic; farewell/loss.
Semiperipheral codes	acceptance of ‘little man’ status; transposition to the afterlife.
*Csak átutazó vagyok itt a földön* (1957/Age 58) — *I’m Just a Passenger on Earth*	
Life-event link	Resident pianist at Kispipa; Sovietized cultural policy; post-’56 years of resigned reflection.
Key lines	“I do not expect miracles from life”; “nothing is ever enough”; “We are all passersby”.
Explicit negation	do not expect; nothing is ever (enough).
Implicit negation	“passerby/sojourner”; “the light goes out”.
Agency – active	“I spend [my days] cheerfully”; normative framing: “if we do it well”.
Agency – constraint	life as transit (limited control).
Communion – presence	collective ‘we’.
Communion – absence	—
Tone / plot	stoic/ironic mixed; quest/journey with a shade of loss.
Semiperipheral codes	impermanence, lack of stability.
*Életemben csak két nőt szerettem* (1962/Age 63) — *I’ve Only Loved Two Women*	
Life-event link	Late years; emotional distance from wife; impending retirement and existential anxiety.
Key lines	“I have no restful nights”; “she left forever”; “I walk the cemetery”; “nothing remained but a grave mound”.
Explicit negation	there is no; ‘not … only’ construction; “did not complain”.
Implicit negation	bereavement/lack, “forever”.
Agency – active	minimal.
Agency – constraint	“I sought the opposite of that” (repeated failure).
Communion – presence	mother and lover as emotional anchors.
Communion – absence	loss of both; visiting the cemetery.
Tone / plot	elegiac/finalistic; loss.
Semiperipheral codes	enduring loss; weak social embeddedness.

*Note*. All song lyrics were downloaded from Zeneszoveg.hu (2025).

Likewise, Table 3 provides examples of the characteristics of narrative tone and plot. The narrative voice is predominantly tragic/finalistic, which is connected to the sense of loss. Affiliative counter-tones occur only sporadically. The lyrics consistently show a closing tendency, which calls forth codes characteristic of the semiperiphery — lack, instability, vulnerability.

## Discussion

As a result of integrating the Kézdi–Wallerstein–McAdams theories, we gain insight into several further connections. First, that high negation density covaries with a finalistic character of the narrative tone. Second, that the two songs with high negation density mentioned above (*Nobody Has Ever Loved Me*, *Just Drink, Drink*) are tied to significant negative life events. Furthermore, that implicit negations, although not grammatical negations, nonetheless signal a narrowing of possibilities. The co-movement of high negation density and constrained agency (“must,” “cannot,” “I cannot”) regularly yields a loss narrative; in other words, a redemptive narrative is rare. The absence of communion (nobody, alone, abandonment) likewise reinforces this and also well represents semiperipheral lack. *Let’s Love Each Other, Children* — a commissioned, positive-toned song — and the collective “we” voice of *I’m Just a Passenger on Earth* add further nuance to the Seress narrative.

### Definition of the Tragic Semiperipheral Self

In the case of the tragic semiperipheral self, the interaction between person and context shows a characteristic pattern. On the semiperiphery, the negative code offers narrative schemata characterized by a persistently negative tone, which makes it more likely that the narrative will not be redemptive and narrows the room for agency and communion; that is, it results in low and defensive agency and communion framed as loss and lack, ultimately producing a finalistic self-narration. The possibility of an alternative self-interpretation becomes smaller the more strongly cultural and structural markers operate in the individual’s life.

### Contemporary Reception as Mirror

The key motifs of Seress’s songs were fate, destiny, and rejection, which may have been connected with his historically and structurally rooted isolation and marginalization. As a self-taught composer of Jewish origin who could not read sheet music, he suffered from the lack of recognition and from not being able to step out of the role of the “little man”. This image already appears in contemporary sources: “…and who could possibly list how many nationally popular songs he composed — yet night after night he plays the piano here, providing the music in this few-square-meter, dimly lit tavern… his fee: a few pengő and a small serving of stew for dinner,” they wrote about him in *Gong* in 1935 (*as quoted* *in* Müller, 1983, p. 8). Memoirs decades later reflect the same image back to Seress: “a small, inconspicuous little man” (*Népszabadság*, 1968, p. 12), “a strongly localized figure” who “almost never crossed the boundaries of District VII” and who lived and created within the inner world of the Jewish petit bourgeoisie of Pest. It was Seress’s choice to identify with this position: in this way he could authentically express the feelings of the “average person,” which was an advantage especially during socialism, but could also elicit the audience’s sympathy in times of crisis. According to Merton’s (1948) theory of the self-fulfilling prophecy, a false definition of a situation can elicit behaviors that make the false situation come true. The legend woven around *Gloomy Sunday* — the “song of suicides” — as a stigma shaped Seress’s self-image and may even have contributed to Seress’s suicide.

### Practical Implications and Pedagogical Reflection

Through a multi-perspectival reading of Seress’s inner development and suicide — starting from cultural, structural, and personal narrative — lessons can also be articulated that may be relevant even today. Our examples reflect on the connections between cultural and structural codes and the personal narrative; our aim is to develop cultural and structural code-awareness, particularly in higher education.

Kézdi’s negative-code theory points out that negation — especially multiple negations — can signal the absence of possibilities and the restriction of alternatives, which may appear as finality in the personal narrative. Members of the cultural–linguistic community may, by decoding these signs, experience their scope for action as limited. A thorough knowledge of the codes, however, opens space for reflection and reframing.

Similarly, insight into resource scarcity arising from a semiperipheral position, the background of vulnerability, and the operation of the capitalist world-system can also affect the shaping of personal narratives. In both examples, useful strategies may include: expanding the time horizon, practicing an active agency-language, and strengthening relational embeddedness. These strategies can also be applied in educational institutions through already proven tools (e.g., reflective journals, relational maps, etc.). In the Hungarian educational environment, the application of code-awareness, knowledge of the relationship between agency and language, and the support of relational embeddedness are also warranted because they promote resilience.

Language, by shaping what and how we reflect on the world, imposes limits on agency.

### Conclusions

Our aim in this study was to interpret Seress’s suicide by integrating Kézdi’s negative code, Wallerstein’s semiperipheral framework, and McAdams’s theory of narrative identity — reading the song lyrics as self-narration. We examined the relations between the “suicide song” myth and Seress’s personal narrative, with particular attention to the tension between his aversion to the stigma of the suicide song and his own suicide. Based on Table 2, negation density peaks in two songs (*Engem még nem szeretett senki*, 1930; *Csak inni, inni*, 1940), which have a finalistic narrative tone. According to Table 3, in these texts the markers of agency are minimal/defensive, while communion is consistently framed as absence. Both songs are linked to life events in which historical and personal crises are simultaneously present. Contemporary reception consistently fixed Seress in the position of the “little man”; the continuous performance of *Gloomy Sunday* turned the myth into a lasting interpretive frame that fed back into his self-narration (internalization), while negotiation was possible only in limited ways.

Within the integrated framework, we identified the co-occurrence of multiple negation (negative code), a finalistic plot, and limited/defensive agency (in the McAdamsian sense). Following Wallerstein, semiperipheral resource scarcity and gatekeeping explain the persistence of institutional marginality; from McLean & Syed’s perspective, in encounters with master narratives internalization appears to dominate negotiation. These processes almost invisibly incorporated not only the Gloomy Sunday myth but also the losing national perspective, which further narrowed the structure of opportunities (opportunity structure). On this basis we proposed the analytic concept of the tragic semiperipheral self (TSS) as a strongly context-bound self-type.

According to our results, although Seress strives to distance himself from the “suicide song” label, he cannot durably detach himself from it. The room for maneuver is narrow: cultural and social constraints are further narrowed by contemporary ideology and politics (antisemitic legislation, the Holocaust). Agency thus appears mostly in defensive form and moving on a narrow track. Methodologically, we combined a qualitative hermeneutic approach with a negation-density analysis, supported by an audit trail and reflexive memos; in this way we contributed to the literature on non-WEIRD psychobiography from an integrative (cultural–semiperipheral–narrative) point of departure. In the future, it would be worthwhile to expand the text corpus (additional song lyrics and press interviews), and — examining other semiperipheral artists — to compare redemptive and tragic patterns.

Seress’s case shows that under the pressure of a semiperipheral master narrative and the negative code — where agency is defensive and communion is written mostly as absence — the songs become at once mirror and shaping force: they give voice to collective suffering while reinforcing a narrative that closes meaning-making in a finalistic manner.
